# Poly[[hemi-μ_4_-oxalato-hemi-μ_2_-oxalato-bis­(μ_3_-pyrazine-2-carboxyl­ato)neodymium(III)silver(I)] monohydrate]

**DOI:** 10.1107/S160053680902128X

**Published:** 2009-06-27

**Authors:** Tian-Jun Feng, Yan-Mei Wen

**Affiliations:** a College of Mathematics, Physics and Software Engineering, Lanzhou Jiaotong University, Lanzhou 730070, People’s Republic of China; b College of Science, Guangdong Ocean University, Zhanjiang 524088, People’s Republic of China

## Abstract

In the title coordination polymer, {[AgNd(C_5_H_3_N_2_O_2_)_2_(C_2_O_4_)]·H_2_O}_*n*_, the Nd^III^ atom is coordinated in a distorted monocapped square-anti­prismatic geometry by two O and two N atoms of two *N*,*O*-bidentate pyrazine-2-carboxyl­ate (2-pzc) ligands, four O atoms of two bidentate oxalate ligands, and one O atom of a monodentate carboxyl­ate group of a 2-pzc ligand. The Ag^I^ ion is coordinated in a distorted tetra­hedral geometry by two N atoms from two monodentate 2-pzc ligands, one O atom from one monodentate oxalate ligand and one O atom of a bridging carboxyl­ate group of a 2-pzc ligand. The oxalate anions link neighbouring neodymium(III) metal centres into Nd–oxalate chains, which are inter­connected by Ag(2-pyz)_2_ units, forming a three-dimensional polymeric framework. Inter­molecular O—H⋯O and C—H⋯O hydrogen bonds are observed in the crystal structure.

## Related literature

For general background to coordination polymers and open-framework materials, see: Barbour (2006 ▶); Kepert (2006 ▶); Kong *et al.* (2008 ▶); Zhang *et al.* (2005 ▶); Gheorghe *et al.* (2002 ▶). For the synthesis and crystal structure of heterometallic complexes of pyrazine-2-carboxylic acid, see: Ciurtin *et al.* (2002 ▶); Dong *et al.* (2000 ▶).
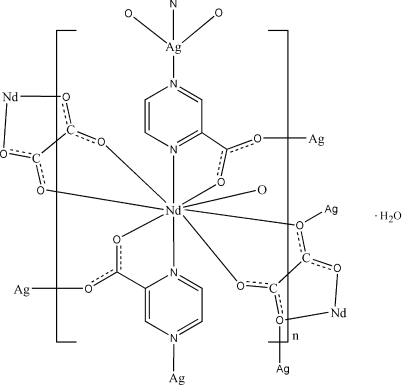


## Experimental

### 

#### Crystal data

[AgNd(C_5_H_3_N_2_O_2_)_2_(C_2_O_4_)]·H_2_O
*M* *_r_* = 604.33
Monoclinic, 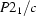

*a* = 10.112 (2) Å
*b* = 18.847 (4) Å
*c* = 8.0359 (16) Å
β = 95.47 (3)°
*V* = 1524.6 (5) Å^3^
*Z* = 4
Mo *K*α radiation
μ = 4.72 mm^−1^
*T* = 293 K
0.32 × 0.26 × 0.21 mm

#### Data collection

Rigaku/MSC Mercury CCD diffractometer
Absorption correction: multi-scan (*REQAB*; Jacobson, 1998 ▶) *T*_min_ = 0.241, *T*_max_ = 0.370
12072 measured reflections
2747 independent reflections
1946 reflections with *I* > 2σ(*I*)
*R*_int_ = 0.093

#### Refinement

*R*[*F* ^2^ > 2σ(*F* ^2^)] = 0.038
*wR*(*F* ^2^) = 0.111
*S* = 1.13
2747 reflections
244 parameters
3 restraints
H-atom parameters constrained
Δρ_max_ = 1.59 e Å^−3^
Δρ_min_ = −1.77 e Å^−3^

### 

Data collection: *RAPID-AUTO* (Rigaku, 1998 ▶); cell refinement: *RAPID-AUTO*; data reduction: *CrystalStructure* (Rigaku/MSC, 2002 ▶); program(s) used to solve structure: *SHELXS97* (Sheldrick, 2008 ▶); program(s) used to refine structure: *SHELXL97* (Sheldrick, 2008 ▶); molecular graphics: *ORTEPII* (Johnson, 1976 ▶); software used to prepare material for publication: *SHELXL97*.

## Supplementary Material

Crystal structure: contains datablocks I, global. DOI: 10.1107/S160053680902128X/rz2330sup1.cif

Structure factors: contains datablocks I. DOI: 10.1107/S160053680902128X/rz2330Isup2.hkl

Additional supplementary materials:  crystallographic information; 3D view; checkCIF report

## Figures and Tables

**Table 1:** Hydrogen-bond geometry (Å, °)

*D*—H⋯*A*	*D*—H	H⋯*A*	*D*⋯*A*	*D*—H⋯*A*
O1*W*—H1*W*⋯O4^i^	0.84	2.31	2.975 (13)	137
C3—H3⋯O1^ii^	0.93	2.34	3.221 (12)	158
C3—H3⋯O8^ii^	0.93	2.49	3.150 (13)	128
C4—H4⋯O5	0.93	2.54	3.170 (13)	125
C9—H9⋯O2^iii^	0.93	2.47	3.270 (15)	145
C9—H9⋯O7^iv^	0.93	2.35	2.971 (15)	124

Symmetry codes: (i) 
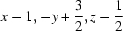
; (ii) 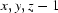; (iii) 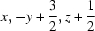; (iv) 
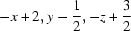
.

**Fig. 1.**
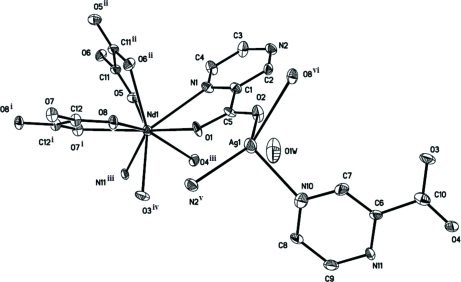
The asymmetric unit of the title compound. Displacement ellipsoids are drawn at the 50% probability level. H atoms are omitted for clarity. [Symmetry codes: (i) 1 - x, 2 - y, 1 - z; (ii) 1 - x, 2 - y, -z; (iii) -1 + x,1.5 - y, -1/2 + z; (iv) -1 + x, y, z; (v) x, y, 1 + z; (vi) 2 - x, 2 - y, 1 - z.]

**Fig. 2.**
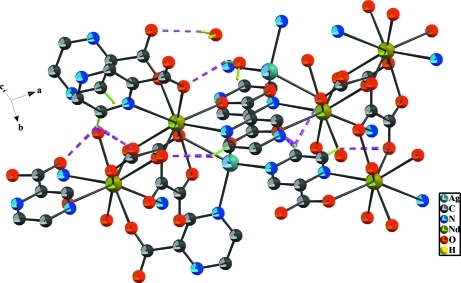
A partial packing diagram of the title compound showing the intermolecular hydrogen bonds as dashed lines. Hydrogen atoms not involved in hydrogen bonds are omitted.

## supplementary crystallographic information

### Comment

In recent years, much research work has been focused on the design and synthesis of new potentially multifunctional heterometallic materials with useful structural properties, such as porosity, gas storage abilities and ion exchange capabilities (Barbour, 2006; Kepert, 2006; Kong *et al.*, 2008; Zhang *et al.*, 2005; Gheorghe *et al.*, 2002). Pyrazine-2-carboxylate (2-pzc) is such a potential multidentate ligand, which can be used to generate high-dimensional heterometallic frameworks (Ciurtin *et al.*, 2002; Dong *et al.*, 2000). On the basis of above considerations, we chose pyrazine-2-carboxylic acid, mixed 4d-4f metal ions and nitric acid as our building blocks. A new three-dimensional 4d-4f coordination framework resulted from the hydrothermal treatment of Nd_2_O_3_, AgNO_3_, oxalic acid, pyrazine-2-carboxylic acid and nitric acid in water.

As depicted in Fig. 1, the asymmetric unit of the title compound contains one neodymium(III) atom, one silver(I) atom, two crystallogaphically independent 2-pzc ligands, two halves of oxalate anions and one lattice water molecule. The neodymium(III) atom is nine-coordinated in a distorted monocapped square-antiprismatic geometry by two O and two N atoms of two N,O-bidentate pyrazine-2-carboxylate (2-pzc) ligands, four O atoms of two bidentate oxalate ligands, and one O atom of a monodentate carboxylate group of a 2-pzc ligand. Each silver(I) ion can be described as having a distorted tetrahedral coordination geometry provided by two N atoms from two monodentate 2-pzc ligands, one O atom from one monodentate oxalate ligand and one O atom of a bridging carboxylate group of a 2-pzc ligand. In the crystal structure, zigzag Nd–oxalate chains are formed *via* the oxalate ligands, with Nd···Nd separations of 6.290 (2) Å and 6.435 (3) Å. The interconnection of the Nd–oxalate chains and Ag(2-pyz)_2_ units result in the formation of a three-dimensional polymeric structure. Intermolecular O—H···O and C—H···O hydrogen bonds involving the non-coordinated water molecules are observed in the crystal structure (Table 1, Fig. 2).

### Experimental

A mixture of Nd_2_O_3_ (0.168 g, 0.5 mmol), AgNO_3_ (0.169 g, 1 mmol), pyrazine-2-carboxylic acid (0.124 g, 1 mmol), oxalic acid (0.09 g, 1 mmol), HNO_3_ (0.12 ml) and H_2_O (10 ml) was placed in a 23 ml Teflon-lined reactor, which was heated to 433 K for 3 d and then cooled to room temperature at a rate of 10 K h^-1^. The colourless crystals obtained were washed with water and dried in air (yield 46% based on Nd).

### Refinement

C-bound H atoms were placed at calculated positions and were treated as riding on their parent atoms, with C—H = 0.93 Å and with *U*_iso_(H) = 1.2 *U*_eq_(C). The water H-atoms were located in a difference map, and were refined with a distance restraint of O—H = 0.84 Å and with *U*_iso_(H) = 1.5 *U*_eq_(O). The hightest peak is located 1.31 Å from O1 and the deepest hole is located 0.94 Å from Nd1.

### Crystal data

**Table:**

[AgNd(C_5_H_3_N_2_O_2_)_2_(C_2_O_4_)]·H_2_O	*F*(000) = 1148
*M**_r_* = 604.33	*D*_x_ = 2.633 Mg m^−^^3^
Monoclinic, *P*2_1_/*c*	Mo *K*α radiation, λ = 0.71073 Å
Hall symbol: -P 2ybc	Cell parameters from 2433 reflections
*a* = 10.112 (2) Å	θ = 1.4–28.0°
*b* = 18.847 (4) Å	µ = 4.72 mm^−^^1^
*c* = 8.0359 (16) Å	*T* = 293 K
β = 95.47 (3)°	Block, colourless
*V* = 1524.6 (5) Å^3^	0.32 × 0.26 × 0.21 mm
*Z* = 4	

### Data collection

**Table:**

Rigaku/MSC Mercury CCD diffractometer	2747 independent reflections
Radiation source: fine-focus sealed tube	1946 reflections with *I* > 2σ(*I*)
graphite	*R*_int_ = 0.093
ω scans	θ_max_ = 25.2°, θ_min_ = 3.3°
Absorption correction: multi-scan (REQAB; Jacobson, 1998)	*h* = −12→12
*T*_min_ = 0.241, *T*_max_ = 0.370	*k* = −22→22
12072 measured reflections	*l* = −9→9

### Refinement

**Table:**

Refinement on *F*^2^	Primary atom site location: structure-invariant direct methods
Least-squares matrix: full	Secondary atom site location: difference Fourier map
*R*[*F*^2^ > 2σ(*F*^2^)] = 0.038	Hydrogen site location: inferred from neighbouring sites
*wR*(*F*^2^) = 0.111	H-atom parameters constrained
*S* = 1.13	*w* = 1/[σ^2^(*F*_o_^2^) + (0.0182*P*)^2^ + 17.3537*P*] where *P* = (*F*_o_^2^ + 2*F*_c_^2^)/3
2747 reflections	(Δ/σ)_max_ = 0.028
244 parameters	Δρ_max_ = 1.59 e Å^−^^3^
3 restraints	Δρ_min_ = −1.77 e Å^−^^3^

### Special details

**Table:**

Geometry. All e.s.d.'s (except the e.s.d. in the dihedral angle between two l.s. planes) are estimated using the full covariance matrix. The cell e.s.d.'s are taken into account individually in the estimation of e.s.d.'s in distances, angles and torsion angles; correlations between e.s.d.'s in cell parameters are only used when they are defined by crystal symmetry. An approximate (isotropic) treatment of cell e.s.d.'s is used for estimating e.s.d.'s involving l.s. planes.
Refinement. Refinement of *F*^2^ against ALL reflections. The weighted *R*-factor *wR* and goodness of fit *S* are based on *F*^2^, conventional *R*-factors *R* are based on *F*, with *F* set to zero for negative *F*^2^. The threshold expression of *F*^2^ > σ(*F*^2^) is used only for calculating *R*-factors(gt) *etc*. and is not relevant to the choice of reflections for refinement. *R*-factors based on *F*^2^ are statistically about twice as large as those based on *F*, and *R*- factors based on ALL data will be even larger.

### Fractional atomic coordinates and isotropic or equivalent isotropic displacement parameters (Å^2^)

**Table:**

	*x*	*y*	*z*	*U*_iso_*/*U*_eq_	
Ag1	1.15900 (9)	0.92327 (6)	0.60684 (11)	0.0369 (3)	
C1	0.9481 (10)	0.9114 (6)	0.0822 (13)	0.026 (3)	
N1	0.8209 (8)	0.9101 (5)	0.0274 (10)	0.027 (2)	
Nd1	0.64656 (5)	0.89514 (3)	0.26047 (7)	0.02128 (18)	
O1	0.8807 (6)	0.9057 (5)	0.3541 (8)	0.0298 (19)	
O1W	0.9655 (10)	0.7408 (7)	0.0126 (18)	0.099 (5)	
H1W	0.9199	0.7431	0.0945	0.149*	
H2W	0.9262	0.7594	−0.0734	0.149*	
C2	1.0449 (10)	0.9146 (6)	−0.0258 (13)	0.029 (3)	
H2	1.1337	0.9136	0.0168	0.035*	
N2	1.0149 (9)	0.9190 (5)	−0.1900 (11)	0.030 (2)	
O2	1.0988 (7)	0.9106 (5)	0.3253 (8)	0.035 (2)	
C3	0.8869 (11)	0.9176 (7)	−0.2454 (13)	0.037 (3)	
H3	0.8622	0.9195	−0.3598	0.045*	
O3	1.6618 (7)	0.8233 (4)	0.5173 (9)	0.034 (2)	
C4	0.7896 (11)	0.9134 (7)	−0.1356 (12)	0.034 (3)	
H4	0.7006	0.9130	−0.1775	0.041*	
O4	1.7098 (7)	0.7193 (4)	0.6421 (10)	0.0317 (18)	
C5	0.9806 (10)	0.9093 (6)	0.2720 (13)	0.027 (3)	
O5	0.5044 (7)	0.9079 (4)	−0.0030 (9)	0.0309 (18)	
O6	0.3693 (7)	0.9833 (4)	−0.1510 (9)	0.0304 (19)	
O7	0.5658 (7)	1.0571 (4)	0.6552 (9)	0.0274 (18)	
O8	0.6685 (7)	0.9827 (4)	0.4928 (8)	0.0270 (18)	
N10	1.2894 (9)	0.8232 (6)	0.6898 (12)	0.039 (3)	
C11	0.4622 (10)	0.9689 (6)	−0.0439 (13)	0.026 (3)	
N11	1.4588 (8)	0.7064 (5)	0.7235 (10)	0.024 (2)	
C12	0.5689 (10)	1.0120 (6)	0.5441 (11)	0.022 (2)	
C6	1.4972 (10)	0.7674 (6)	0.6640 (13)	0.027 (3)	
C7	1.4155 (11)	0.8253 (6)	0.6552 (14)	0.031 (3)	
H7	1.4498	0.8685	0.6234	0.037*	
C8	1.2491 (11)	0.7609 (6)	0.7402 (15)	0.034 (3)	
H8	1.1617	0.7560	0.7654	0.041*	
C9	1.3340 (11)	0.7018 (7)	0.7569 (14)	0.033 (3)	
H9	1.3016	0.6587	0.7922	0.040*	
C10	1.6348 (11)	0.7698 (7)	0.6033 (13)	0.030 (3)	

### Atomic displacement parameters (Å^2^)

**Table:**

	*U*^11^	*U*^22^	*U*^33^	*U*^12^	*U*^13^	*U*^23^
Ag1	0.0338 (5)	0.0520 (7)	0.0262 (4)	0.0081 (4)	0.0096 (4)	0.0043 (4)
C1	0.028 (6)	0.024 (7)	0.026 (6)	−0.007 (5)	0.006 (5)	−0.004 (5)
N1	0.028 (5)	0.035 (6)	0.018 (4)	−0.008 (4)	0.002 (4)	0.001 (4)
Nd1	0.0222 (3)	0.0239 (3)	0.0182 (3)	0.0008 (3)	0.0041 (2)	−0.0008 (3)
O1	0.009 (3)	0.063 (6)	0.017 (3)	−0.001 (4)	0.001 (3)	0.002 (4)
O1W	0.052 (7)	0.092 (11)	0.155 (12)	0.000 (6)	0.020 (7)	−0.042 (9)
C2	0.019 (5)	0.040 (8)	0.028 (6)	0.009 (5)	0.003 (4)	0.009 (5)
N2	0.032 (5)	0.037 (6)	0.022 (5)	−0.001 (4)	0.008 (4)	0.004 (4)
O2	0.021 (4)	0.066 (7)	0.017 (4)	0.004 (4)	−0.004 (3)	−0.006 (4)
C3	0.033 (7)	0.064 (10)	0.014 (5)	0.016 (6)	−0.003 (5)	−0.007 (5)
O3	0.036 (5)	0.040 (6)	0.027 (4)	−0.009 (4)	0.005 (3)	0.015 (4)
C4	0.036 (7)	0.055 (9)	0.012 (5)	−0.002 (6)	0.007 (5)	−0.007 (5)
O4	0.034 (4)	0.015 (4)	0.048 (5)	−0.001 (3)	0.013 (4)	0.008 (4)
C5	0.027 (6)	0.029 (7)	0.025 (5)	−0.008 (5)	0.010 (5)	0.006 (5)
O5	0.039 (5)	0.029 (5)	0.025 (4)	−0.004 (4)	0.000 (3)	0.003 (4)
O6	0.022 (4)	0.033 (5)	0.035 (4)	0.005 (3)	−0.007 (3)	0.002 (4)
O7	0.024 (4)	0.033 (5)	0.024 (4)	−0.001 (3)	0.000 (3)	−0.008 (4)
O8	0.026 (4)	0.034 (5)	0.021 (4)	0.007 (3)	0.006 (3)	−0.007 (3)
N10	0.029 (5)	0.043 (7)	0.043 (6)	0.004 (5)	−0.001 (5)	0.004 (5)
C11	0.024 (6)	0.028 (7)	0.027 (6)	0.002 (5)	0.003 (5)	0.008 (5)
N11	0.018 (4)	0.027 (6)	0.029 (5)	0.000 (4)	0.010 (4)	0.001 (4)
C12	0.034 (6)	0.020 (6)	0.012 (5)	0.000 (5)	0.003 (4)	−0.012 (4)
C6	0.026 (6)	0.032 (7)	0.023 (5)	0.000 (5)	0.002 (4)	0.013 (5)
C7	0.034 (7)	0.016 (7)	0.043 (7)	−0.001 (5)	0.003 (5)	0.002 (5)
C8	0.033 (6)	0.022 (7)	0.050 (7)	−0.004 (5)	0.019 (6)	0.011 (6)
C9	0.038 (7)	0.029 (7)	0.032 (6)	−0.004 (5)	0.006 (5)	0.011 (5)
C10	0.044 (7)	0.029 (8)	0.019 (5)	−0.007 (6)	0.010 (5)	−0.004 (5)

### Geometric parameters (Å, °)

**Table:**

Ag1—N2^i^	2.291 (9)	C3—H3	0.9300
Ag1—O2	2.299 (7)	O3—C10	1.267 (13)
Ag1—N10	2.360 (10)	O3—Nd1^vii^	2.461 (7)
C1—N1	1.320 (13)	C4—H4	0.9300
C1—C2	1.370 (14)	O4—C10	1.240 (14)
C1—C5	1.530 (14)	O4—Nd1^viii^	2.465 (7)
N1—C4	1.319 (13)	O5—C11	1.259 (13)
N1—Nd1	2.707 (8)	O6—C11	1.242 (12)
Nd1—O1	2.423 (6)	O6—Nd1^ii^	2.455 (8)
Nd1—O6^ii^	2.455 (8)	O7—C12	1.235 (11)
Nd1—O5	2.456 (7)	O7—Nd1^v^	2.482 (7)
Nd1—O3^iii^	2.461 (7)	O8—C12	1.252 (12)
Nd1—O4^iv^	2.465 (8)	N10—C8	1.319 (15)
Nd1—O7^v^	2.482 (7)	N10—C7	1.332 (14)
Nd1—O8	2.486 (7)	C11—C11^ii^	1.53 (2)
Nd1—N11^iv^	2.692 (9)	N11—C9	1.317 (13)
O1—C5	1.260 (12)	N11—C6	1.318 (14)
O1W—H1W	0.8400	N11—Nd1^viii^	2.692 (9)
O1W—H2W	0.8405	C12—C12^v^	1.57 (2)
C2—N2	1.328 (13)	C6—C7	1.367 (15)
C2—H2	0.9300	C6—C10	1.517 (15)
N2—C3	1.329 (13)	C7—H7	0.9300
N2—Ag1^vi^	2.291 (9)	C8—C9	1.406 (16)
O2—C5	1.231 (12)	C8—H8	0.9300
C3—C4	1.385 (15)	C9—H9	0.9300
			
N2^i^—Ag1—O2	124.8 (3)	N2—C2—H2	119.2
N2^i^—Ag1—N10	98.4 (3)	C1—C2—H2	119.2
O2—Ag1—N10	106.5 (3)	C2—N2—C3	117.0 (9)
N1—C1—C2	121.5 (10)	C2—N2—Ag1^vi^	127.6 (7)
N1—C1—C5	116.3 (9)	C3—N2—Ag1^vi^	115.3 (7)
C2—C1—C5	122.3 (9)	C5—O2—Ag1	120.1 (7)
C1—N1—C4	117.7 (9)	N2—C3—C4	121.1 (10)
C1—N1—Nd1	116.7 (6)	N2—C3—H3	119.5
C4—N1—Nd1	125.5 (7)	C4—C3—H3	119.5
O1—Nd1—O6^ii^	93.6 (3)	C10—O3—Nd1^vii^	153.6 (7)
O1—Nd1—O5	137.2 (2)	N1—C4—C3	121.1 (10)
O6^ii^—Nd1—O5	65.6 (2)	N1—C4—H4	119.4
O1—Nd1—O3^iii^	78.7 (2)	C3—C4—H4	119.4
O6^ii^—Nd1—O3^iii^	144.2 (3)	C10—O4—Nd1^viii^	126.5 (7)
O5—Nd1—O3^iii^	139.7 (3)	O2—C5—O1	128.3 (10)
O1—Nd1—O4^iv^	84.8 (3)	O2—C5—C1	117.1 (9)
O6^ii^—Nd1—O4^iv^	133.5 (3)	O1—C5—C1	114.6 (9)
O5—Nd1—O4^iv^	84.6 (3)	C11—O5—Nd1	118.1 (7)
O3^iii^—Nd1—O4^iv^	81.1 (3)	C11—O6—Nd1^ii^	118.1 (7)
O1—Nd1—O7^v^	136.9 (2)	C12—O7—Nd1^v^	122.0 (6)
O6^ii^—Nd1—O7^v^	74.4 (2)	C12—O8—Nd1	121.6 (6)
O5—Nd1—O7^v^	75.5 (2)	C8—N10—C7	114.8 (10)
O3^iii^—Nd1—O7^v^	87.6 (3)	C8—N10—Ag1	128.1 (8)
O4^iv^—Nd1—O7^v^	133.3 (2)	C7—N10—Ag1	116.0 (8)
O1—Nd1—O8	72.4 (2)	O6—C11—O5	126.5 (10)
O6^ii^—Nd1—O8	69.4 (2)	O6—C11—C11^ii^	117.6 (13)
O5—Nd1—O8	125.8 (3)	O5—C11—C11^ii^	115.8 (11)
O3^iii^—Nd1—O8	75.0 (3)	C9—N11—C6	116.9 (9)
O4^iv^—Nd1—O8	149.6 (2)	C9—N11—Nd1^viii^	127.6 (7)
O7^v^—Nd1—O8	64.6 (2)	C6—N11—Nd1^viii^	115.5 (6)
O1—Nd1—N11^iv^	138.7 (3)	O7—C12—O8	128.2 (10)
O6^ii^—Nd1—N11^iv^	127.1 (2)	O7—C12—C12^v^	116.3 (11)
O5—Nd1—N11^iv^	68.0 (3)	O8—C12—C12^v^	115.5 (10)
O3^iii^—Nd1—N11^iv^	71.9 (3)	N11—C6—C7	121.2 (10)
O4^iv^—Nd1—N11^iv^	62.7 (3)	N11—C6—C10	117.0 (10)
O7^v^—Nd1—N11^iv^	70.6 (3)	C7—C6—C10	121.9 (10)
O8—Nd1—N11^iv^	124.5 (2)	N10—C7—C6	123.6 (11)
O1—Nd1—N1	61.6 (2)	N10—C7—H7	118.2
O6^ii^—Nd1—N1	71.3 (3)	C6—C7—H7	118.2
O5—Nd1—N1	76.1 (2)	N10—C8—C9	122.1 (11)
O3^iii^—Nd1—N1	130.2 (3)	N10—C8—H8	118.9
O4^iv^—Nd1—N1	67.3 (3)	C9—C8—H8	118.9
O7^v^—Nd1—N1	142.1 (3)	N11—C9—C8	121.1 (11)
O8—Nd1—N1	115.7 (3)	N11—C9—H9	119.5
N11^iv^—Nd1—N1	119.8 (3)	C8—C9—H9	119.5
C5—O1—Nd1	130.6 (6)	O4—C10—O3	126.2 (11)
H1W—O1W—H2W	111.8	O4—C10—C6	117.0 (10)
N2—C2—C1	121.5 (10)	O3—C10—C6	116.8 (10)

Symmetry codes: (i) *x*, *y*, *z*+1; (ii) −*x*+1, −*y*+2, −*z*; (iii) *x*−1, *y*, *z*; (iv) *x*−1, −*y*+3/2, *z*−1/2; (v) −*x*+1, −*y*+2, −*z*+1; (vi) *x*, *y*, *z*−1; (vii) *x*+1, *y*, *z*; (viii) *x*+1, −*y*+3/2, *z*+1/2.

### Hydrogen-bond geometry (Å, °)

**Table:**

*D*—H···*A*	*D*—H	H···*A*	*D*···*A*	*D*—H···*A*
O1W—H1W···O4^iv^	0.84	2.31	2.975 (13)	137
C3—H3···O1^vi^	0.93	2.34	3.221 (12)	158
C3—H3···O8^vi^	0.93	2.49	3.150 (13)	128
C4—H4···O5	0.93	2.54	3.170 (13)	125
C9—H9···O2^ix^	0.93	2.47	3.270 (15)	145
C9—H9···O7^x^	0.93	2.35	2.971 (15)	124

Symmetry codes: (iv) *x*−1, −*y*+3/2, *z*−1/2; (vi) *x*, *y*, *z*−1; (ix) *x*, −*y*+3/2, *z*+1/2; (x) −*x*+2, *y*−1/2, −*z*+3/2.
